# Enantioselective radical chemistry: a bright future ahead

**DOI:** 10.3762/bjoc.21.174

**Published:** 2025-10-28

**Authors:** Anna C Renner, Sagar S Thorat, Hariharaputhiran Subramanian, Mukund P Sibi

**Affiliations:** 1 Department of Chemistry and Biochemistry, North Dakota State University, Fargo, North Dakota, 58105-5516, USA

**Keywords:** chiral Lewis acid, electrochemistry, enantioselective radical reaction, organocatalysis, photoenzymatic catalysis, photoredox

## Abstract

This perspective is focused on enantioselective free radical reactions. It describes several important catalytic asymmetric strategies applied to enantioselective radical reactions, including chiral Lewis acid catalysis, organocatalysis, photoredox catalysis, chiral transition-metal catalysis and photoenzymatic catalysis. The application of electrochemistry to asymmetric radical transformations is also discussed.

## Introduction

Asymmetric catalysis plays an integral role in the enantioselective synthesis of organic compounds. A wide variety of enantioenriched catalysts ranging from chiral organometallic complexes to organocatalysts (small organic molecules) have been designed, synthesized, and successfully used in several organic transformations [1–3]. Despite these advances, catalytic methods involving radical intermediates were very rare until the 1990s. Since then, meticulous research by several research groups has led to significant advances in this area [4–8]. This perspective focuses on several important contributions to the science of asymmetric radical reactions. Pioneering work on chiral Lewis acid catalysis and iminium catalysis is discussed initially. This is followed by the recently emerging areas of transition-metal catalysis, photoenzymatic catalysis, and electrochemistry.

## Perspective

### Radical generation and reactions

Synthetic methods based on free radical chemistry are some of the most efficient and powerful tools for the construction of carbon–carbon and carbon–heteroatom bonds. Unlike many ionic reactions, radical reactions are often functional group tolerant and carried out under mild, neutral conditions, avoiding harsh acidic or basic conditions that can promote epimerization or decomposition of the product. Additionally, radical chemistry offers opportunities to achieve transformations that may not proceed via two-electron processes.

**Figure 1:**
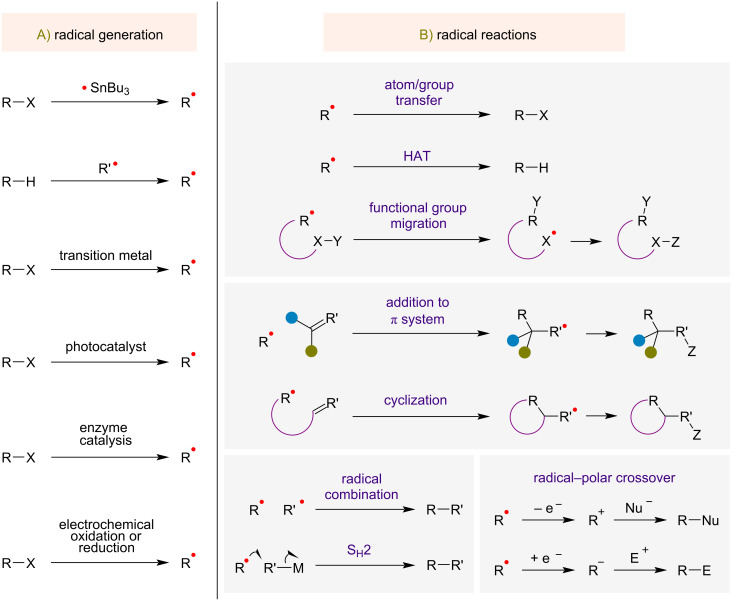
Methods of radical generation (A) and general types of radical reactions (B).

Radicals can be generated through several different approaches, summarized in Figure 1A. The use of organostannanes to generate carbon-centered radicals was formerly commonplace but has been largely supplanted by greener methods employing less-toxic reagents. Using alternative methods, radicals can be generated by hydrogen atom transfer (HAT), resulting in the homolytic cleavage of a carbon–hydrogen bond. Other approaches for radical generation in modern radical transformations include the use of transition metals or photoredox catalysts. In photoredox catalysis, radical generation often involves single-electron transfer (SET) to or from a photoexcited state of a photoredox catalyst, usually a metal complex or organic molecule. Two other notable strategies for radical generation are photoenzymatic catalysis and electrochemical oxidation or reduction.

Free radicals can undergo several types of basic reactions (Figure 1B), including atom or group transfer, addition to a π-bond, and radical–radical combination. In an atom or group transfer reaction, an atom or group is transferred to the radical. Important processes of this kind are hydrogen atom transfer (HAT) and halogen atom abstraction. An intramolecular group transfer reaction can result in the net migration of a functional group. Addition of a radical to a π-bond (carbon–carbon or carbon–heteroatom) is another common radical reaction and can occur either intermolecularly or as an intramolecular cyclization reaction. Radical coupling or combination is a possible transformation that is more feasible when one of the radicals is relatively stabilized (a persistent radical). Trapping a radical with a transition metal is one way to convert a free radical to a more stable intermediate, which can subsequently undergo coupling with another radical via an S_H_2 (bimolecular homolytic substitution) mechanism. Lastly, some noteworthy radical processes proceed through a radical–polar crossover pathway, in which one-electron oxidation or reduction of the radical yields a cationic or anionic intermediate that participates in a subsequent step through a polar mechanism. An important aspect of many of these radical reactions is that they can result in the formation of new carbon–carbon bonds, a fundamental goal in organic synthesis.

### Strategies for asymmetric radical reactions

Stereoselectivity in radical reactions can be challenging to control. Many radicals are highly reactive, and radicals moreover have typically low inversion barriers, resulting in no permanent chirality at the radical center. Stereochemistry in radical reactions is generally governed by the subsequent reaction with a radical trap. Most enantioselective radical strategies developed thus far include the incorporation of a chiral element into the radical species or the radical trap (either stoichiometrically or catalytically). Chiral transition-metal catalysis affords the dual advantages of introducing stereochemical discrimination on both the radical and the trap via chiral ligands.

Early research on parameters governing stereoselectivity in radical reactions was achieved with the help of radicals or radical traps appended with chiral auxiliaries. The research on auxiliary-based chiral Lewis acid catalysis inspired Porter, Sibi and others to transpose the concept to radical chemistry. A large number of enantioselective radical reactions that were reported during 1996–2007 were mainly based on chiral Lewis acid-mediated/catalyzed free radical reactions.

The past three decades have seen enormous advances in the development of enantioselective radical reactions, particularly using organocatalysts. Some of the notable chiral organocatalysts imparting enantioselectivity include chiral secondary amines, chiral Brønsted acids, and chiral H-bonding catalysts. The drawbacks of chiral Lewis acids have been overcome to an extent using organocatalysis. The use of photochemistry to generate radicals by light-induced electron transfer has resulted in elegant enantioselective radical transformations. Several transition-metal photocatalysts [9] and organo-photocatalysts [10–12] have been successfully incorporated into enantioselective radical reactions.

The use of transition metals to catalyze enantioselective radical reactions can be considered a major advancement in the field of asymmetric catalysis. Several metals such as cobalt, nickel, copper, and titanium have been employed successfully to catalyze enantioselective radical reactions. Two earth abundant transition metals that have found extensive application in enantioselective radical reactions are copper and nickel. These metals, particularly Ni, can be used in radical–radical coupling reactions which are not possible in traditional organocatalyzed reactions.

**Figure 2:**
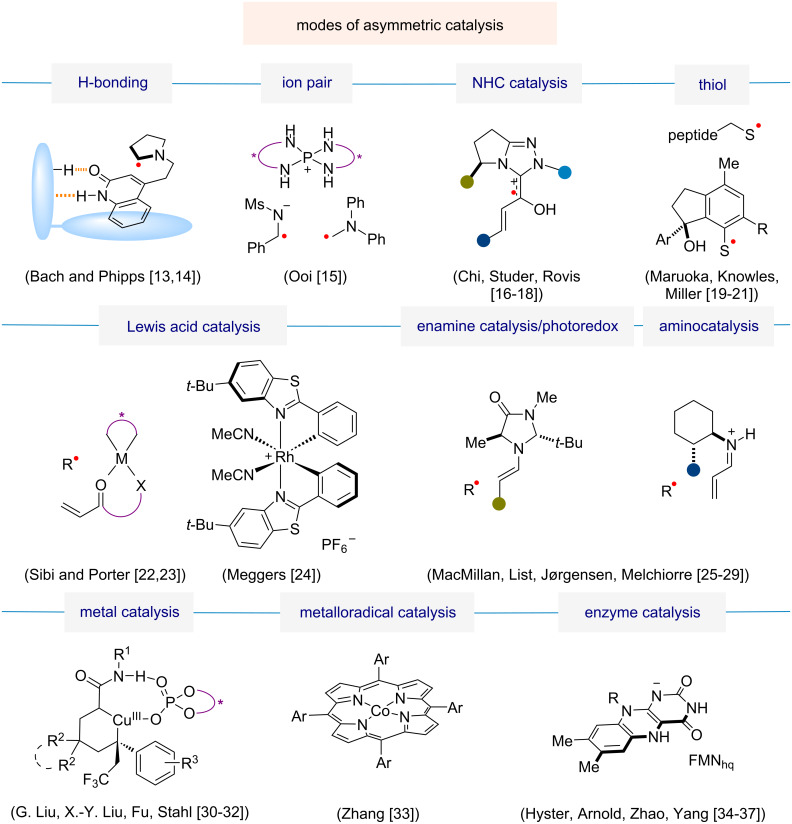
Chiral catalysis in enantioselective radical chemistry [13–37].

An overview of different modes of asymmetric catalysis in radical chemistry is presented in Figure 2. Of these, this perspective focuses on Lewis acid catalysis, organocatalysis (including enamine catalysis), photoredox catalysis, transition-metal catalysis, and enzyme catalysis. Progress in enantioselective reactions using these approaches is discussed with the help of one or two examples in each category to highlight the outstanding achievements in the past three decades. Other modes of catalysis relying on hydrogen-bonding [13–14], ion pairs [15], *N*-heterocyclic carbene (NHC) catalysts [16–18], or thiols [19–21] are not covered here but can also be effective for achieving high levels of enantioselectivity in radical reactions.

### Lewis acid-catalyzed radical reactions

**Scheme 1:**
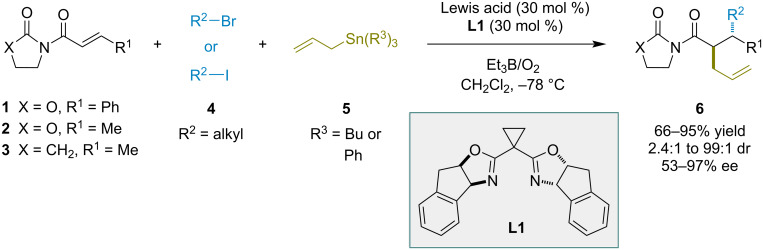
Diastereo- and enantioselective additions of nucleophilic radicals to *N*-enoyloxazolidinone and pyrrolidinone electrophiles.

In the context of enantioselective radical reactions, initial examples of catalytic methodologies were based on chiral Lewis acid catalysis, with catalysts used in stoichiometric or sub-stoichiometric amounts [38–39]. Porter and Sibi disclosed the first enantioselective examples of conjugate additions to electron-deficient olefins by nucleophilic radicals [22–23]. In these studies, alkyl radicals underwent conjugate addition to *N*-enoyloxazolidinones. Sibi later described enantioselective tandem radical conjugate addition–trapping reactions that formed two carbon–carbon bonds and created two vicinal stereocenters in a single transformation (Scheme 1) [40]. The reactions were catalyzed by chiral Lewis acids and involved conjugate addition of a nucleophilic alkyl radical to an α,β-unsaturated substrate containing an oxazolidinone or pyrrolidinone template. The resulting α-radical was trapped with an allylstannane and the addition and trapping occurred in an *anti* fashion. Products were obtained with up to 97% ee via catalysis by complexes of magnesium or copper(II) with ligand **L1**. The absolute stereochemistry of the product could be controlled by a simple change from copper(II) to magnesium Lewis acids while using the same chiral ligand, thus obviating the need for both enantiomers of the ligand.

Although chiral Lewis acid-mediated radical reactions were groundbreaking, they suffered from major disadvantages: (a) high catalyst loadings (stoichiometric or sub-stoichiometric), (b) large amounts of the radical initiator, (c) the need for a large excess of radical precursor, (d) use of toxic H-atom sources such as tin hydride, and (e) limited variation in the nature of the radicals (mostly nucleophilic).

### Organocatalyzed radical reactions

Chiral secondary amine-based catalytic systems have been used in several asymmetric transformations [41–42]. MacMillan obtained chiral free radicals by stoichiometric single electron transfer (SET) oxidation of enamines, formed by the reaction between chiral secondary amines and aldehydes. This mode of activation was called SOMO (singly occupied molecular orbital) catalysis and was employed in several organic transformations [43–48].

**Scheme 2:**
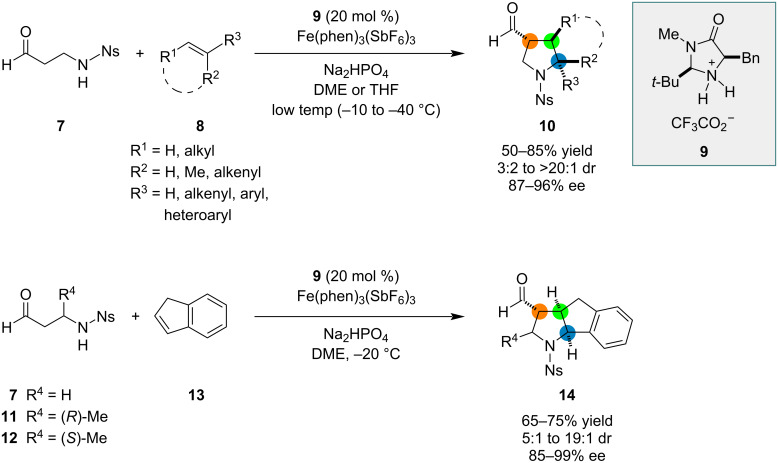
Organocatalyzed formal [3 + 2] cycloadditions affording substituted pyrrolidines.

Using SOMO catalysis, MacMillan and co-workers developed a method for the synthesis of substituted pyrrolidines from β-aminoaldehydes and olefins in a formal [3 + 2] cycloaddition (Scheme 2) [44]. The transformation was proposed to proceed via a radical–polar crossover mechanism involving single-electron oxidation of an enamine intermediate, addition of the resulting radical to the olefin, single-electron oxidation of the adduct to form a carbocationic intermediate, and intramolecular nucleophilic attack on the carbocation to form the pyrrolidine ring. The reaction tolerated a range of substituents on the olefin, giving the products with high enantioselectivity. Reactions of chiral β-methyl-substituted aldehydes **11** and **12** with indene (**13**) yielded tricyclic products with good diastereoselectivity and particularly high optical purity (99% ee).

**Scheme 3:**
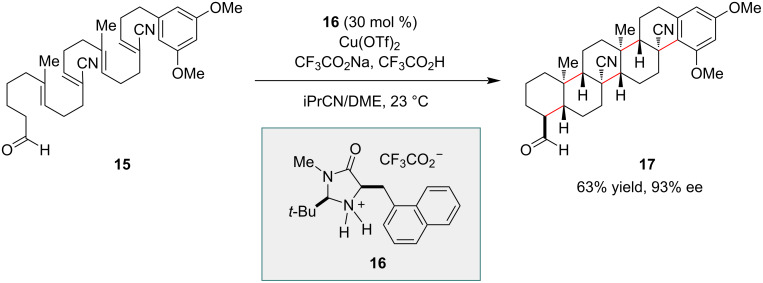
Synthesis of a hexacyclic compound via an organocatalyzed enantioselective polyene cyclization.

Organo-SOMO catalysis was applied in the development of enantioselective polyene cyclizations, which demonstrated the power of the catalytic strategy. In the presence of a chiral amine catalyst **16** (Scheme 3) and the mild oxidant Cu(OTf)_2_, polyenes with a terminal aldehyde group underwent intramolecular cyclizations affording polycyclic products in a highly diastereo- and enantioselective manner [45]. The polyene substrates were designed to facilitate a radical cyclization process, with alternating electron-poor and electron-rich olefins so as to enable polarity matching of the olefins and radical intermediates. With this design, a relatively electrophilic radical could readily add to a nearby electron-rich olefin, and the resulting nucleophilic radical could add to an electron-poor olefin (e.g., substituted with a cyano group). In one example, polyene **15** underwent cyclization to afford hexacyclic product **17** with 93% ee in a single process that established five new carbon–carbon bonds and nine contiguous stereocenters, including four all-carbon quaternary centers (Scheme 3).

### Transition-metal-catalyzed radical reactions

Recent advances in enantioselective radical reactions catalyzed by base metals (e.g., Fe, Co, Ni, Mn) should enable the development of methodologies that expand radical transformations to a larger number of substrates in green and sustainable ways.

Nickel is an earth-abundant transition metal that has been used in several organic transformations [49–52]. Chiral nickel catalysts have been demonstrated to forge C(sp^2^)–C(sp^2^) as well as aliphatic C(sp^3^)–C(sp^3^) bonds.

**Scheme 4:**
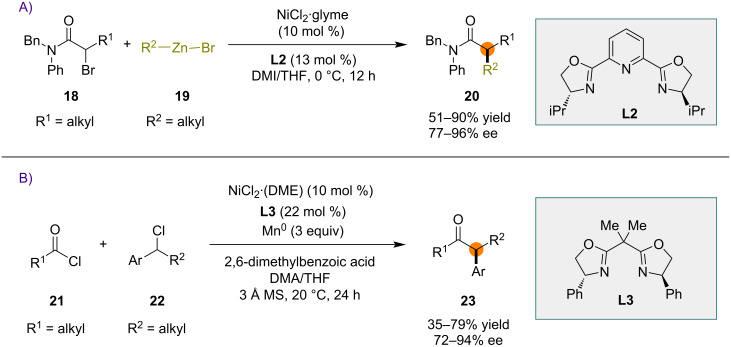
Nickel-catalyzed asymmetric cross-coupling reactions.

The Fu group reported a nickel-catalyzed α-alkylation of racemic secondary α-bromoamides **18** using organozinc reagents **19** (Scheme 4A) [32]. A chiral nickel complex, obtained from the mixture of chiral pyridinebisoxazoline ligand **L2** and NiCl_2_·diglyme, was used as the catalyst to obtain the coupling products **20** in good yield and high enantioselectivity.

The first nickel-catalyzed asymmetric reductive cross-coupling reaction between acid chlorides **21** and secondary benzylic chlorides **22** was reported by Reisman and co-workers (Scheme 4B) [53]. A catalytic system consisting of a combination of chiral bisoxazoline **L3** and NiCl_2_·(DME) was able to catalyze the reaction between **21** and **22** in the presence of a stoichiometric amount of Mn as reductant. α,α-Disubstituted ketones **23** were obtained in moderate to good yields and up to 94% ee.

A mechanistically distinct way of achieving enantioselective radical transformations is the use of metalloradical catalysis [54]. A metalloradical is a persistent metal-centered radical species that can homolytically activate substrates without other initiators, light, or electricity. Several different transition-metal complexes have been employed in metalloradical catalysis, including porphyrin complexes of cobalt(II) [55–56] and iron(III) [57–58]. Both cobalt in oxidation state +2 and iron in oxidation state +3 can be viewed as persistent metalloradicals.

**Scheme 5:**
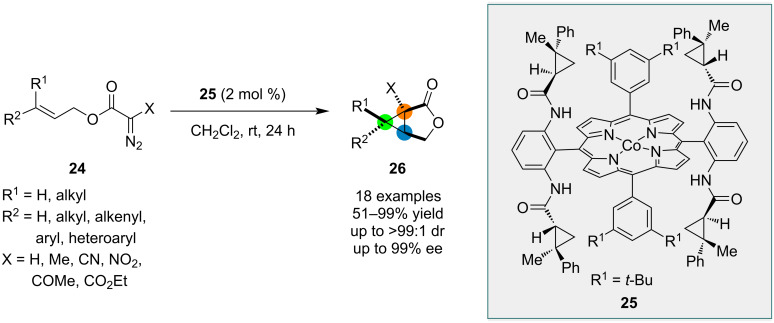
Chiral cobalt–porphyrin metalloradical-catalyzed radical cyclization reactions.

Zhang and co-workers reported a unique chiral cobalt–porphyrin complex **25** that could catalyze the conversion of diazoester **24** to lactone **26** (Scheme 5) [33]. In most cases, lactone **26** was formed with complete diastereocontrol and excellent enantioselectivity. A mechanism involving an initial reaction between Co(II) metalloradical **25** and diazoester **24** to furnish a Co(III)-bonded α-ester radical (α-Co(III)-ester radical) with extrusion of nitrogen was proposed by the authors. Further steps include (a) *5-exo* cyclization by α-Co(III)-ester radical and (b) homolytic substitution at the carbon atom by 3-*exo-tet-*cyclization to generate bicyclic lactone **26**.

The Zhang group has extended the chiral metalloradical catalysis to cyclopropanation by the intermolecular reaction between styrenes and ketodiazoacetates [59]. Cyclopropanes were obtained in good yields with high relative and absolute stereocontrol.

Properties, such as relatively high earth abundance, low cost, less toxicity, and existence of multiple oxidation states (I, II, and III) make copper an essential transition metal for catalytic purposes [60–61]. The fact that copper, like other transition metals, has multiple oxidation states makes it an excellent redox catalyst. Owing to the reducing ability of copper(I), substrates can be reduced to radicals and oxidize copper(I) to copper(II) in the process. Copper(II) can oxidize other organic radicals to cations by SET or reversibly react with other radicals to form copper(III) species that can undergo fundamental organometallic steps such as reductive elimination [62].

G. Liu and Stahl disclosed an elegant methodology to functionalize benzylic C–H bonds via copper catalysis [30]. Under the catalytic conditions, alkylarenes were converted to benzylic nitriles in good yields and excellent enantioselectivities. In the proposed mechanism, a chiral Cu(III) complex forms from an achiral benzylic radical and a Cu(II)–CN species, and subsequent reductive elimination affords the enantioenriched benzylic nitrile product.

Recently, X.-Y. Liu and co-workers reported an enantioselective aminofluoroalkylation of alkenes using a Cu(I)/chiral phosphoric acid catalytic system [31]. Aminoalkenes react with Togni’s reagent in the presence of a catalytic amount of CuCl and a chiral phosphoric acid to yield pyrrolidines. Using this methodology, chiral α-quaternary substituted pyrrolidines were synthesized in good yields and excellent enantioselectivities.

**Scheme 6:**
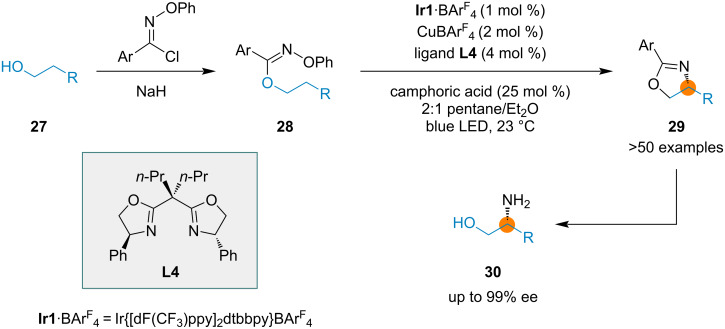
Enantioselective radical chaperone catalysis.

A radical chaperone methodology is based on a multicatalytic system in which a chiral Cu(I) catalyst, Brønsted acid (camphoric acid) and Ir photocatalyst work synergistically. An asymmetric synthetic method based on radical C–H functionalization was reported by Nagib and co-workers for the preparation of chiral β-aminoalcohols [63]. Chiral copper(I) complexes convert imidate radicals, formed transiently through energy-transfer catalysis, to oxazolines. The transformation includes a regioselective and enantioselective HAT process. Upon blue LED irradiation, oxime imidates (derived from alcohols and imidoyl chlorides) in the presence of CuBAr^F^_4_/bisoxazoline **L4** catalyst, an acid co-catalyst, and iridium photocatalyst form chiral oxazolines **29** in good yields and excellent enantioselectivities (82–97% ee) (Scheme 6). Chiral β-aminoalcohols **30** can be obtained by the hydrolysis of **29**.

Another transition metal that has seen extensive use in radical chemistry is titanium. Titanium(III) complexes can function as Lewis acids but can also generate carbon-centered radicals via SET to an organic substrate. In an example from Lin and co-workers, a chiral titanium catalyst promoted enantioselective isomerization reactions of cyclic *meso*-epoxides as part of a bimetallic titanium/cobalt catalytic system [64].

### Photoredox catalysis in radical reactions

The ability of a photocatalyst (organic small molecule or transition-metal complex) to undergo single electron transfer (SET) to a variety of organic substrates or organometallic complexes upon photoexcitation has enabled the synthetic community to access reactive open-shell species under very mild conditions. The ability of excited photocatalysts to induce other reagents, substrates, or other catalysts to participate in new activation modes makes them a powerful tool in organic synthesis [65–70]. Over the years, many catalytic systems were developed by merging photoredox processes and other catalytic modes. Some of the disadvantages of chiral Lewis acid catalysis such as toxic reducing agents, high catalytic loading, and limitations in the type of radicals used can be overcome using photoredox catalysis. Synergistic photoredox and other forms of catalysis (using chiral Lewis acids, chiral Brønsted acids, H-bonding compounds, polarity reversal catalysts, etc.) expanded the scope of radical traps used and introduced alternate ways of quenching, in some cases involving radical–polar crossover processes. The photoredox catalysts used in most of the synergistic catalysis are Ir and Ru-based systems that are expensive and less readily available. This limitation can be overcome by developing green and sustainable organophotoredox systems.

**Scheme 7:**
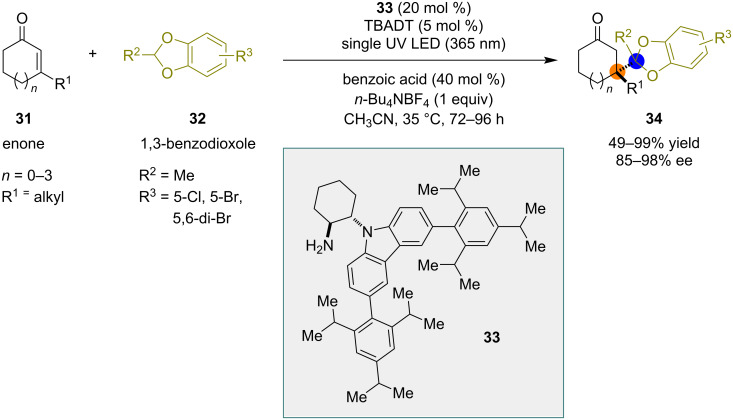
Enantioselective radical addition by decatungstate/iminium catalysis.

Melchiorre and co-workers reported a dual catalytic system that involves photoredox and chiral organocatalysts for the construction of all-carbon quaternary centers. The authors studied radical additions to β,β-disubstituted cyclic enones (Scheme 7) [29]. The dual catalytic system of tetrabutylammonium decatungstate (TBADT) and chiral amine **33** was able to catalyze the reaction between benzodioxoles **32** and cyclic β,β-disubstituted enones **31**. Cyclic ketones **34** bearing all-carbon quaternary stereocenters at the β-position were obtained in good yields and high enantioselectivity.

### Enzyme-catalyzed radical reactions

An emerging trend in the field of enantioselective radical transformations is the use of enzymatic catalysis to control absolute stereoselectivity. Some remarkable transformations have been demonstrated using this promising greener catalysis. This methodology has a bright future when used individually or synergistically with other catalytic systems. Enzymatic catalysis can pose challenges including enzyme engineering, reaction scale-up, etc. but is optimal in terms of the toxicity profile of the reaction conditions.

Biomolecules such as enzymes are attractive candidates for efficient and selective synthesis owing to their unprecedented catalytic activity and selectivity. Advances in the field of protein engineering have made enzymatic catalysis more amenable to enantioselective organic synthesis in the past decade. Recent advances in the development of synergistic catalytic systems, particularly involving photoredox catalysts, have led to the emerging area of photoenzymatic catalysis. Several new modes of activation successfully catalyzed by enzymes have been demonstrated [71–74].

**Scheme 8:**
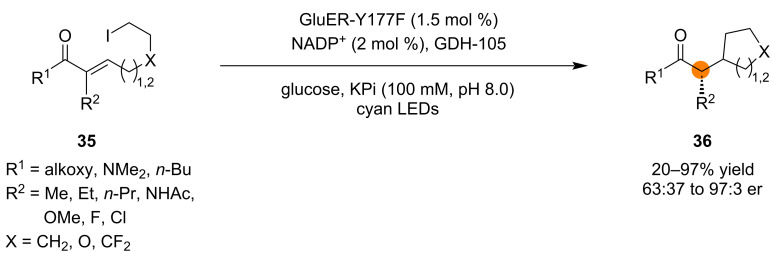
An ene-reductase-catalyzed photoenzymatic enantioselective radical cyclization/enantioselective HAT process.

Notable photoenzymatic reactions involving radical cyclizations have been reported. In one photoenzymatic transformation developed by the Hyster group, α-chloroamides were converted to enantioenriched 5-, 6-, 7-, and 8-membered lactams through an enantioselective radical cyclization followed by a diastereoselective HAT, producing contiguous stereocenters [75]. In the subsequent year, the same group reported intramolecular Giese-type radical cyclization reactions involving unstabilized alkyl radicals generated from alkyl iodides (Scheme 8) [34]. The generation of unstabilized, nucleophilic alkyl radicals from alkyl iodides was more challenging than the generation of more electrophilic alkyl radicals from α-halocarbonyl compounds as in the previous report. In this case, radical formation from the alkyl iodide was accomplished through photoexcitation of a charge-transfer complex of the substrate and flavin in the active site of the enzyme (a mutated ene-reductase). Subsequent 5-*exo*-*trig* or 6-*exo*-*trig* cyclization and HAT furnished the enantioenriched products with up to 97:3 er.

**Scheme 9:**
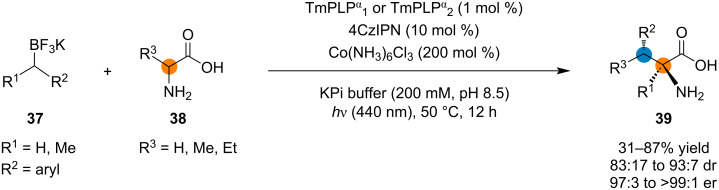
Photoenzymatic oxidative C(sp^3^)–C(sp^3^) coupling reactions between organoboron compounds and amino acids.

In addition to intramolecular radical reactions, intermolecular radical transformations have also been achieved using photoenzymatic catalysis, such as the addition of fluoroalkyl radicals to alkene substrates [36] and cross-electrophile coupling of alkyl halides and nitroalkanes [76]. Yang and co-workers reported photoenzymatic asymmetric C(sp^3^)–C(sp^3^) oxidative cross-couplings between organoboron reagents and amino acids (Scheme 9) [37]. The authors used a synergistic system consisting of an engineered threonine aldolase, a photoredox catalyst, and an oxidizing agent. With this catalytic system, they were able to accomplish the C–H functionalization of glycine and α-branched amino acids, obtaining α-tri- and tetrasubstituted amino acids with moderate to good yields, good diastereoselectivity, and high enantioselectivity.

New catalytic methods have been developed by merging photoenzymatic catalysis with small-molecule photoredox catalysis. Enantioselective radical acylation reactions of *N*-hydroxyphthalimide esters and aldehydes, yielding α-substituted ketones, were catalyzed by a radical acyl transferase (an engineered thiamine diphosphate-dependent lyase) working together with an organic dye (eosin Y) that served as a photoredox catalyst [77]. Highly diastereo- and enantioselective three-component couplings were catalyzed by an evolved pyridoxal decarboxylase and a small-molecule photoredox catalyst (rose bengal), affording diversely substituted products [78].

### Electrochemical radical reactions

The organic chemistry community has witnessed a resurgence of interest in electrochemical methods for organic synthesis [79]. Organic electrochemistry can provide mild reaction conditions, good functional group tolerance, and scalability [80]. Electrochemical methods offer the opportunity to tune reaction conditions by controlling parameters such as potential, current, electrode, and electrolyte [81], providing a sophisticated platform for attaining optimal reactivity. A variety of catalytic strategies can be employed under electrochemical conditions, and many asymmetric electrochemical transformations have been established [82–84]. Electrochemistry is well-suited to applications in one-electron processes, including transformations involving organic radicals, and can obviate the need for a stoichiometric chemical oxidant or reductant [80,85], thereby reducing toxicity and waste. With these advantages, organic electrochemistry holds significant promise for improving efficiency and sustainability while enabling the development of new transformations.

**Scheme 10:**
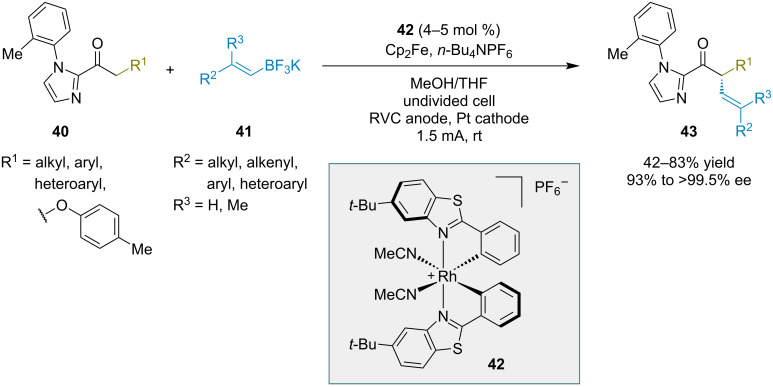
Electrochemical α-alkenylation reactions of 2-acylimidazoles catalyzed by a chiral-at-rhodium Lewis acid.

Meggers and co-workers developed an electrochemical method for the enantioselective α-C(sp^3^) alkenylation of ketones containing imidazole auxiliaries (Scheme 10) [24]. The transformation was catalyzed by a chiral-at-rhodium Lewis acid **42**. A variety of ketone electrophiles **40** and alkenyl trifluoroborate nucleophiles **41** were converted to the corresponding α-alkenylated products **43** with excellent enantioselectivities. Alkenyl trifluoroborates with (*Z*) configuration gave higher product yields than the analogous (*E*)-alkenyl trifluoroborates, but the corresponding alkenyl group in the product had (*E*) configuration in all cases. A key enabling aspect of the method was the use of ferrocene as a redox mediator, which played an important role in preventing substrate decomposition under the electrochemical conditions.

**Scheme 11:**
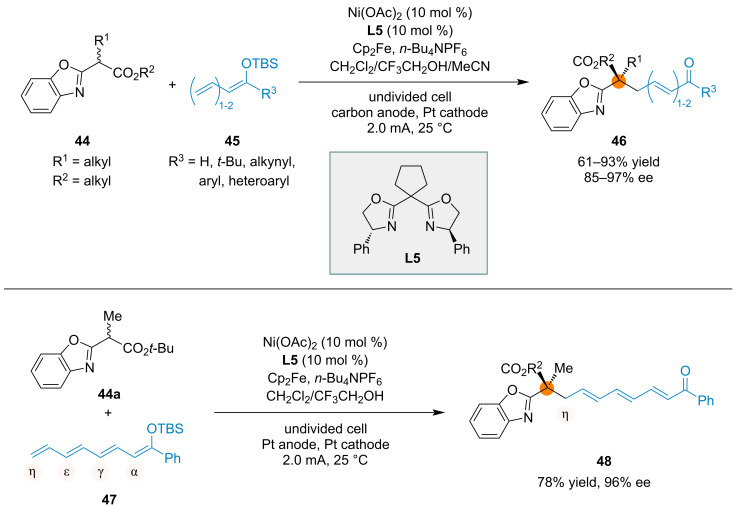
Regio- and enantioselective electrochemical reactions of silyl polyenolates catalyzed by a chiral nickel catalyst.

An electrochemical method enabled regio- and enantioselective radical reactions of silyl polyenolates with racemic α-branched esters, yielding products with a new all-carbon quaternary stereocenter (Scheme 11) [86]. With a Ni(II)–bisoxazoline complex as the catalyst, various substrates underwent the transformation with good to high yields and high enantioselectivities. The reactions were regioselective, resulting in the selective functionalization of the terminal carbon atom of the silyl polyenolate. In the reaction of silyl tetraenol ether **47**, selective reaction at the η position occurred, and the product **48** was obtained in 78% yield and 96% ee.

## Conclusion

This perspective highlights important contributions in the area of enantioselective radical reactions. These examples demonstrate a wide variety of radical transformations, asymmetric catalytic systems, and strategies for radical generation. The selection included here not only highlights outstanding accomplishments but also draws attention to promising areas for future research.

In the past three decades, numerous advances have overcome major disadvantages of early enantioselective radical methods, including the use of tin hydrides as H-atom sources and excess quantities of radical initiator or radical precursor. A broad range of enantioselective radical transformations has been developed, allowing for the asymmetric construction of C(sp^3^)–C(sp^3^), C(sp^3^)–C(sp^2^), and C(sp^3^)–C(sp) bonds and challenging motifs such as vicinal stereocenters and all-carbon quaternary centers. A diverse range of chiral catalysts has been employed to achieve enantioselectivity, including Lewis acids, organocatalysts, transition-metal complexes, and enzymes. In many cases, low catalyst loadings have been achieved, especially in transition-metal catalyzed, photoredox, and biocatalytic methods.

Photoredox catalysis has had a tremendous impact on the field of organic radical chemistry. In many modern radical methods, photoredox catalysis is used in combination with another mode of catalysis, such as organocatalysis, chiral Lewis acid catalysis, or enzymatic catalysis. Photoredox-based methods have introduced new, atom-economical approaches for radical generation, in some cases enabling direct radical generation and C–H functionalization of aliphatic substrates via HAT and thereby avoiding the use of more highly functionalized radical precursors such as organic halides.

The application of electrochemistry for enantioselective radical transformations is a highly promising approach. Electrochemistry provides a way to avoid the use of stoichiometric chemical oxidants or reductants and to achieve new transformations that are highly regio- and stereoselective. Though not covered in this perspective, flow chemistry and mechanochemistry are also increasingly popular areas that hold significant potential for applications in radical chemistry. Compared with conventional batch synthesis, flow chemistry can improve efficiency and facilitate reaction scale-up [87–90]. Mechanochemistry allows reactions to be conducted with no solvent or with only a small volume of added liquid, thus providing a green alternative to solution-phase chemistry [91]. Mechanical grinding or milling also provides a means to generate radicals via piezocatalysis [91], an emerging strategy that warrants investigation in the context of enantioselective radical reactions.

Future research holds the key to addressing current limitations in the scope and capabilities of enantioselective radical chemistry. The existing literature features a predominant use of nucleophilic radicals, a small number of H-atom donors, and limited application of hydrogen-bonding catalysis in radical reactions. Further studies on enantioselective transformations of electrophilic radicals, alternative H-atom sources, and additional modes of catalysis (including hydrogen-bonding catalysis) would be valuable. Additionally, the development of more efficient methods, using lower catalyst loadings, remains an important goal for ongoing research. The use of more sustainable photoredox catalysts – organic molecules or complexes of earth-abundant metals – is a key endeavor toward decreasing reliance on the iridium and ruthenium complexes commonly employed in photoredox methods.

As a whole, asymmetric radical chemistry offers significant utility for synthetic applications and great potential for further development. Building upon the significant advances of the past three decades, future research may overcome existing limitations and deliver more efficient and green methods for enantioselective radical transformations.

## Data Availability

Data sharing is not applicable as no new data was generated or analyzed in this study.
